# Supplementary material for: PARG Protein Regulation Roles in Drosophila Longevity Control
Source: Int J Mol Sci. 2024 Jun 4;25(11):6189. doi: 10.3390/ijms25116189 (PMC11173342; doi:10.3390/ijms25116189)
Supplement: Supplementary file 1 [file ijms-25-06189-s001.zip › ijms-3009636-supplementary.pdf]

SUPPLEMENTARY APPENDIX FOR

PARG Protein Regulation Roles in *Drosophila* Longevity Control

Guillaume Bordet[1], and Alexei V. Tulin[1]*

1 - University of North Dakota, Grand Forks, ND

Address correspondence to:

Email: Alexei.Tulin@und.edu

THIS FILE INCLUDES:

Supplemental Table S1. PARG controls the expression of genes coding for muscle structure component.

Supplemental Table S2. PARG controls the expression of genes involved in cell death processes.

Supplemental Table S3. PARG controls the expression of genes involved in toxin resistance**.**

Supplemental Figure S1: Western blot of pADPr.

Supplemental Figure S2: Western blot for PARG-GFP.

Supplemental Figure S3: Western blot for H3.

Supplemental Figure S4: The absence of PARG C-terminal domains affects the lifespan.

Supplemental Figure S5: pADPr level is increased during aging progression of wild type animals.

D. melanogaster name	Fold change during aging	Fold change in *parg* mutant	Protein group	Human ortholog
Sls	-29.01	2.89	Titin	HMCN1
MLP60A	-20.92	3.73	Z-line associated protein	CSRP3
TPnC25D	-9.97	13.95	Troponin C	CALML6
Salm	-4.9	1.04	Zinc finger transcription factor	SALL1
Eya	-3.44	-1.08	Tyrosine phosphatase	EYA2
TPnC47D	-118	14.18	Troponin C	CALML6
Fln	-85.68	1.80	A band located protein	/
Strn-Mlck	-75.2	1.76	Calcuim/calmodulin-dependent protein kinase	MYLK
Unc-89	-35.71	10.20	obscurin	SPEG
Act88F	-20.58	-1.91	Actin	ACTB
TPnC41C	-19.2	2.71	Troponin C	CALML6
Act79B	-13.2	-1.35	Actin	ACTB
Prm	-3.48	3.25	Paramyosin	/
Mlp84B	-2.79	2.00	Z-line associated protein	CSRP3
Tn	-2.06	-1.34	Z-line associated protein	TRIM2
TPnC4	-6.56	1.22	Troponin C	CALM1
Tm2	-3.65	3.92	Tropomyosin	TPM3
Up	-3.24	4.08	Troponin T	TNNT2
mlc2	-3.2	5.96	Myosin light chain	MYL
Tm1	-3.16	2.35	Tropomyosin	TPM1
Fhos	-3.04	-1.01	Formin like	FHOD3
mlc1	-2.78	6.35	Myosin light chain	MYL
Act87E	-2.75	2.76	Actin	ACTB
mhc	-2.47	11.57	Myosin heavy chain	MYH
Mf	-2.44	2.90	A band located protein	/
Bt	-2.27	5.67	Projectin	TTN
Wupa	-2.24	3.35	Troponin I	TNNI2

Supplemental Table S1: PARG controls the expression of genes coding for muscle structure component. Table showing the list of the muscle structure components (**Column 1**) and how they are misregulated during adult aging process (**Column 2**) or in *parg* mutant during the end of third instar larval stage (**Column 3**). A green label means that the gene is significantly upregulated while a red label means that the gene is significantly downregulated. **Column 4**

displays the main function of the protein and **Column 5** displays the closest Human ortholog. Source data for Aging data is (*1*) while source data for *parg* mutant is (*2*).

D. melanogaster name	Fold change during aging	Fold change in *parg* mutant	Protein group	Human ortholog
Fkh	3,86	1.01	Fork head box transcription factor	FOXA2
Strica	3,84	1.03	Caspase	CASP3
Ubr3	2,37	1.11	RING finger protein	UBR3
Hand	11,25	1.88	bHLH transcription factor	HAND2
Lola	3,33	1.08	C2H2 zinc finger transcription factor	ZBTB20
Naam	3,11	1.91	Nicotinamide amidase	/
Fwe	3,11	-1.23	Other calcium channel-forming subunit	CACFD1
Decay	6,72	2.01	Caspase	CASP3
Damm	13,09	4.74	Caspase	CASP3
Diap1	2,7	-1.16	RING finger protein	BIRC2

Supplemental Table S2: PARG controls the expression of genes involved in cell death processes. Table showing the list of the genes involved in cell death processes (**Column 1**) and how they are misregulated during adult aging process (**Column 2**) or in *parg* mutant during the end of third instar larval stage (**Column 3**). A green label means that the gene is significantly upregulated while a red label means that the gene is significantly downregulated. **Column 4** displays the main function of the protein and **Column 5** displays the closest Human ortholog. Source data for Aging data is (*1*) while source data for *parg* mutant is (*2*).

D. melanogaster name	Fold change during aging	Fold change in *parg* mutant	Function	Human ortholog
Cyp313a1	-16.6	1.81	Unknown	CYP26B1
Cyp4ac2	-7.3	1.36	Toxic chermicals breakdown (predicted)	CYP4V2
Cyp4e1	-5	6.94	Permethrin resistance	CYP4V2
Cyp6a21	-6.2	2.95	Toxic chermicals breakdown (predicted)	CYP3A4
Cyp305a1	-2.7	-1.14	Unknown	CYP2J2
Cyp18a1	-2.2	2.50	Steroid hormone inhibition	CYP2J2
CG34172	-2.9	1.67	Unknown	COX7A1
CG6870	-2.1	-2.00	Unknown	CYB5A
Cyp4g15	2.1	1.56	Toxic chermicals breakdown (predicted)	CYP4V2
Cyp4ac3	8.4	2.35	Toxic chermicals breakdown (predicted)	CYP4V2
Cyp4p1	7.9	1.61	DDT resistance	CYP4V2
Cyp4p3	13.2	6.20	DDT resistance	CYP4V2
Cyp6w1	2.5	15.09	DDT resistance	CYP3A4
Cyp9c1	6.1	2.30	DDT resistance	CYP3A4
Cyp6d2	31	-1.13	Camptothecin resistance	CYP3A4
Cyp28d1	2.6	2.47	Toxic chermicals breakdown (predicted)	CYP3A4
Cyp6d4	3.2	1.30	Toxic chermicals breakdown (predicted)	CYP3A4
Cyp309a1	10.2	1.94	Unknown	CYP3A4
Cyp9b2	2.2	2.24	Toxic chermicals breakdown (predicted)	CYP3A5
Cyp9b1	2.3	9.09	Toxic chermicals breakdown (predicted)	CYP3A5
Cyp9h1	14.1	-1.39	Toxic chermicals breakdown (predicted)	CYP3A5
Cyp6a13	3.7	1.52	Toxic chermicals breakdown (predicted)	CYP3A7
Cyp304a1	53.7	1.32	Toxic chermicals breakdown (predicted)	CYP2F1
Cyp12a5	2.2	4.54	Unknown	CYP24A1
Cyp12e1	4.3	1.97	Unknown	CYP24A1
CG10337	2.2	-1.78	Unknown	CYB561D1
CG13077	11.8	13.09	Unknown	CYB561D1

Supplemental Table S3: PARG controls the expression of genes involved in toxin resistance. Table showing the list of the genes involved in toxin resistance (**Column 1**), a subset of the genes involved in defense response processes, and how they are misregulated during adult

aging process (**Column 2**) or in *parg* mutant during the end of third instar larval stage (**Column 3**). A green label means that the gene is significantly upregulated while a red label means that the gene is significantly downregulated. **Column 4** displays the main function of the protein and **Column 5** displays the closest Human ortholog. Source data for Aging data is (*1*) while source data for *parg* mutant is (*2*).

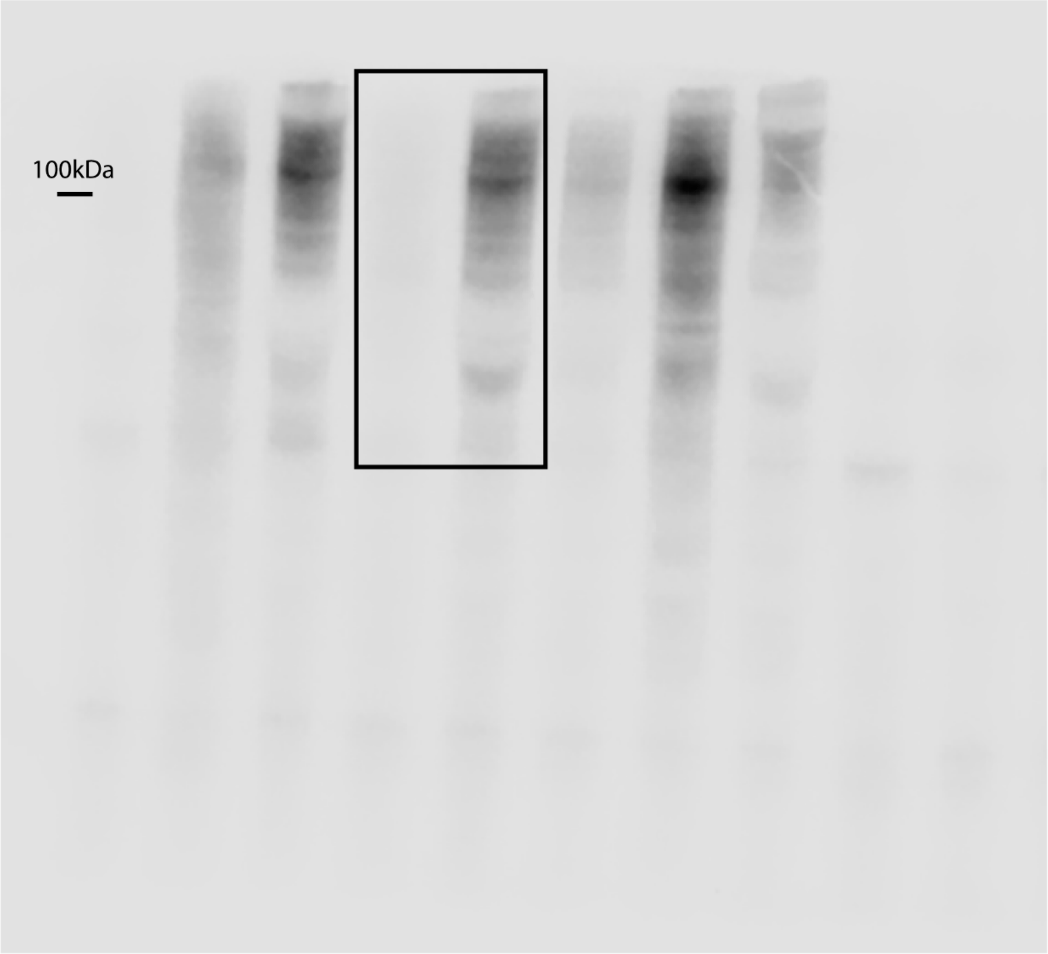

Supplemental Figure S1: Western blot for pADPr. The cropped section presented in Fig.2C is highlighted by the black square.

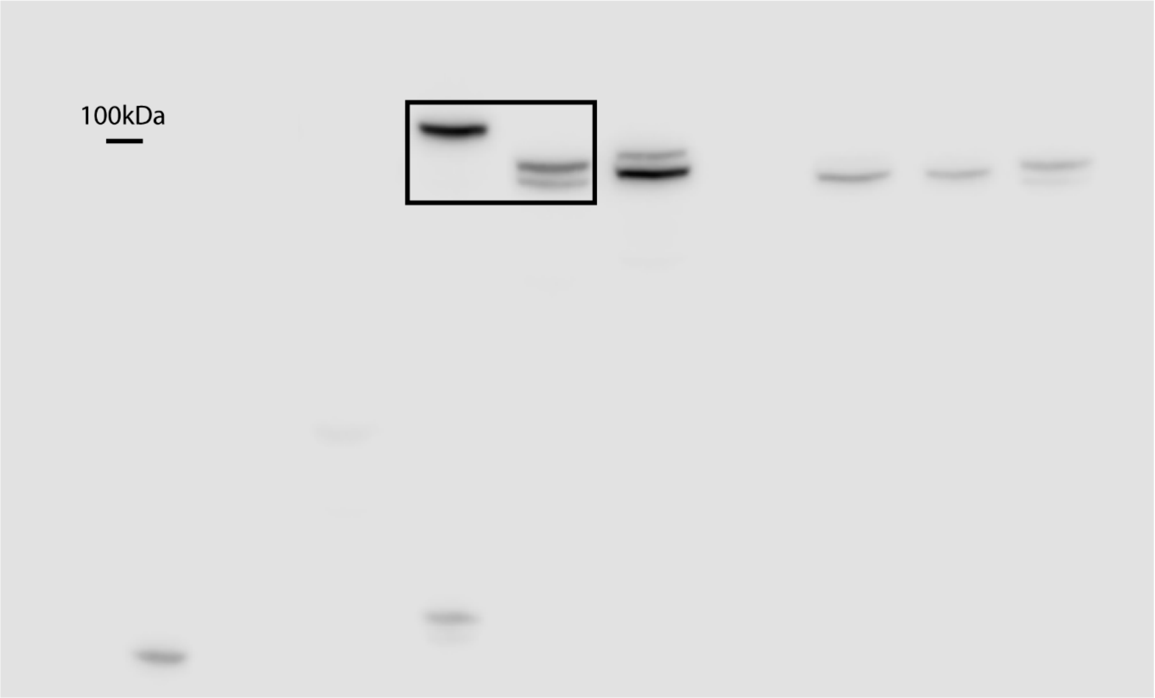

Supplemental Figure S2: Western blot for PARG-GFP. The cropped section presented in Fig.2C is highlighted by the black square.

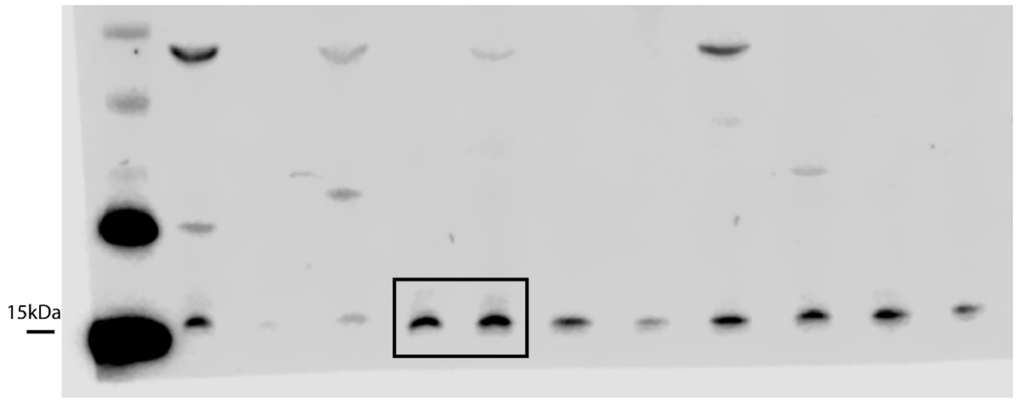

Supplemental Figure S3: Western blot for H3. The cropped section presented in Fig.2C is highlighted by the black square.

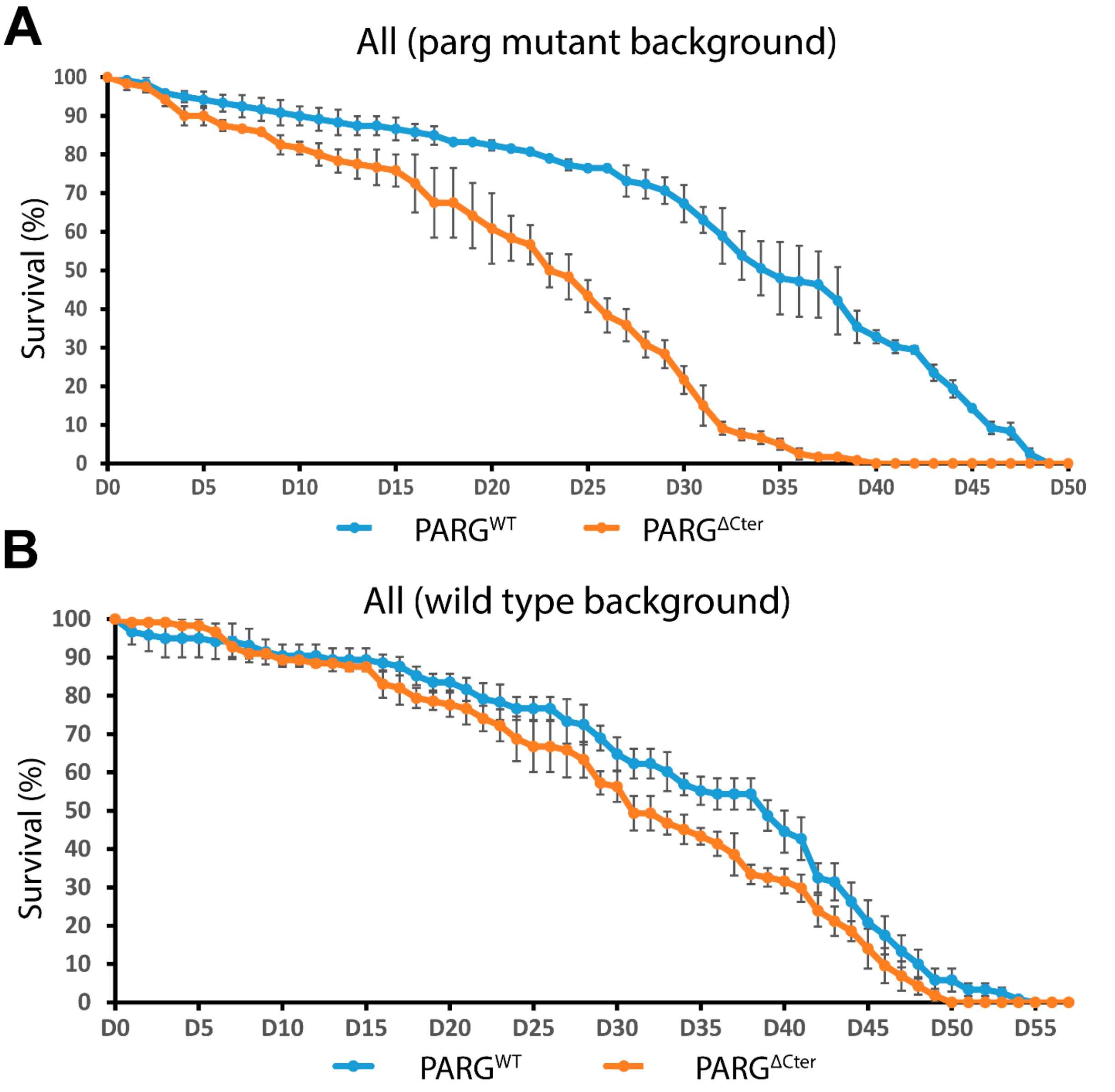

Supplemental Figure S4: The absence of PARG C-terminal domains affects the lifespan.

A-B) Lifespan curves depict adult populations expressing either $PARG^{WT}$ (blue) or $PARG^{\Delta Cter}$ (orange) in a *parg* mutant (**A**) or in a wild type background (**B**). Y-axis represents the percentage of flies surviving on specific days post-hatching, with Day 0 denoting adult emergence. Triplicate experiments were conducted, and error bars represent standard error of mean (SEM).

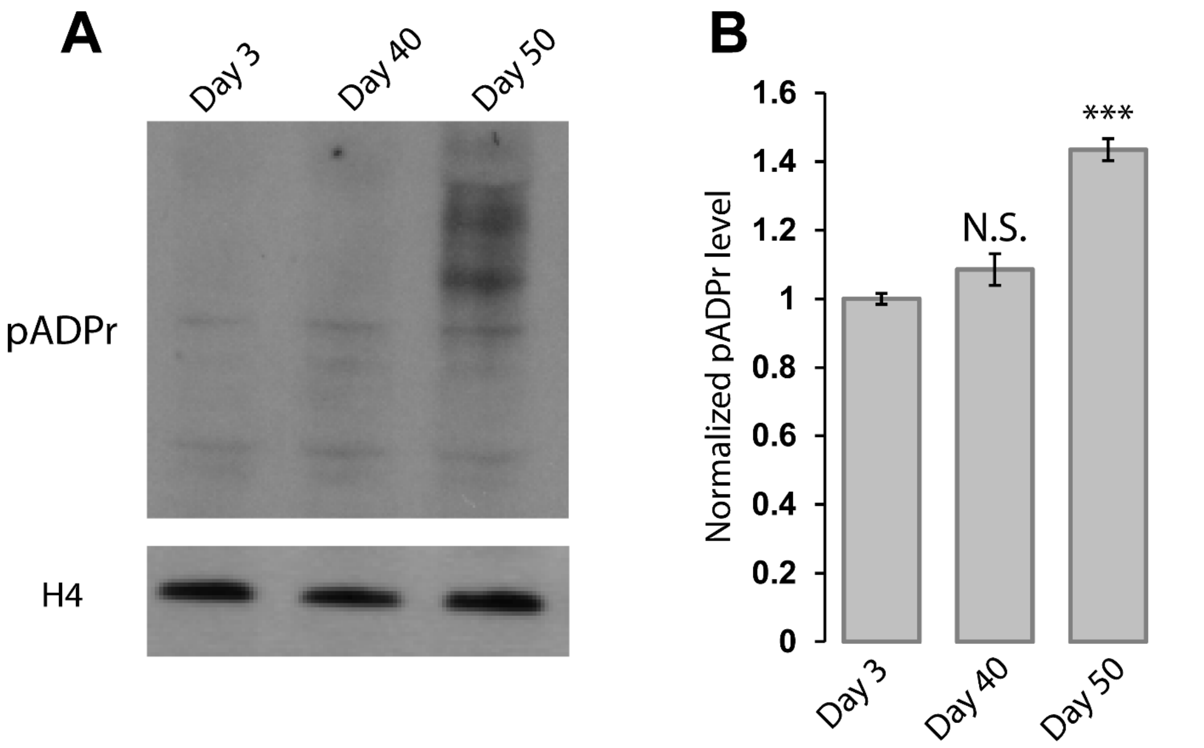

Supplemental Figure S5: pADPr level is increased during aging progression of wild type animals.

A) Western blot showing the level of poly(ADP-ribose) (pADPr) in wild type animals at different timepoints: Day-3, Day-40, and Day-50 old adult flies. H4 is shown as a loading control. **B**) Quantitative assessment of pADPr levels based on two independent blots. Statistical analysis was conducted using an unpaired two-tailed t-test. N.S indicates non-significant results while *** indicates a p-value < 0.01.

REFERENCES

1. Bordet G, Lodhi N, Kossenkov A, Tulin A. Age-Related Changes of Gene Expression Profiles in Drosophila. Genes (Basel). 2021;12(12).
2. Bordet G, Karpova I, Tulin AV. Poly(ADP-ribosyl)ating enzymes cooperate to coordinate development. Sci Rep. 2022;12(1):22120.
